# Supplementary figures and images for: Relationship between Species Richness, Biomass and Structure of Vegetation and Mycobiota along an Altitudinal Transect in the Polar Urals
Source: J Fungi (Basel). 2020 Dec 9;6(4):353. doi: 10.3390/jof6040353 (PMC7770575; doi:10.3390/jof6040353)

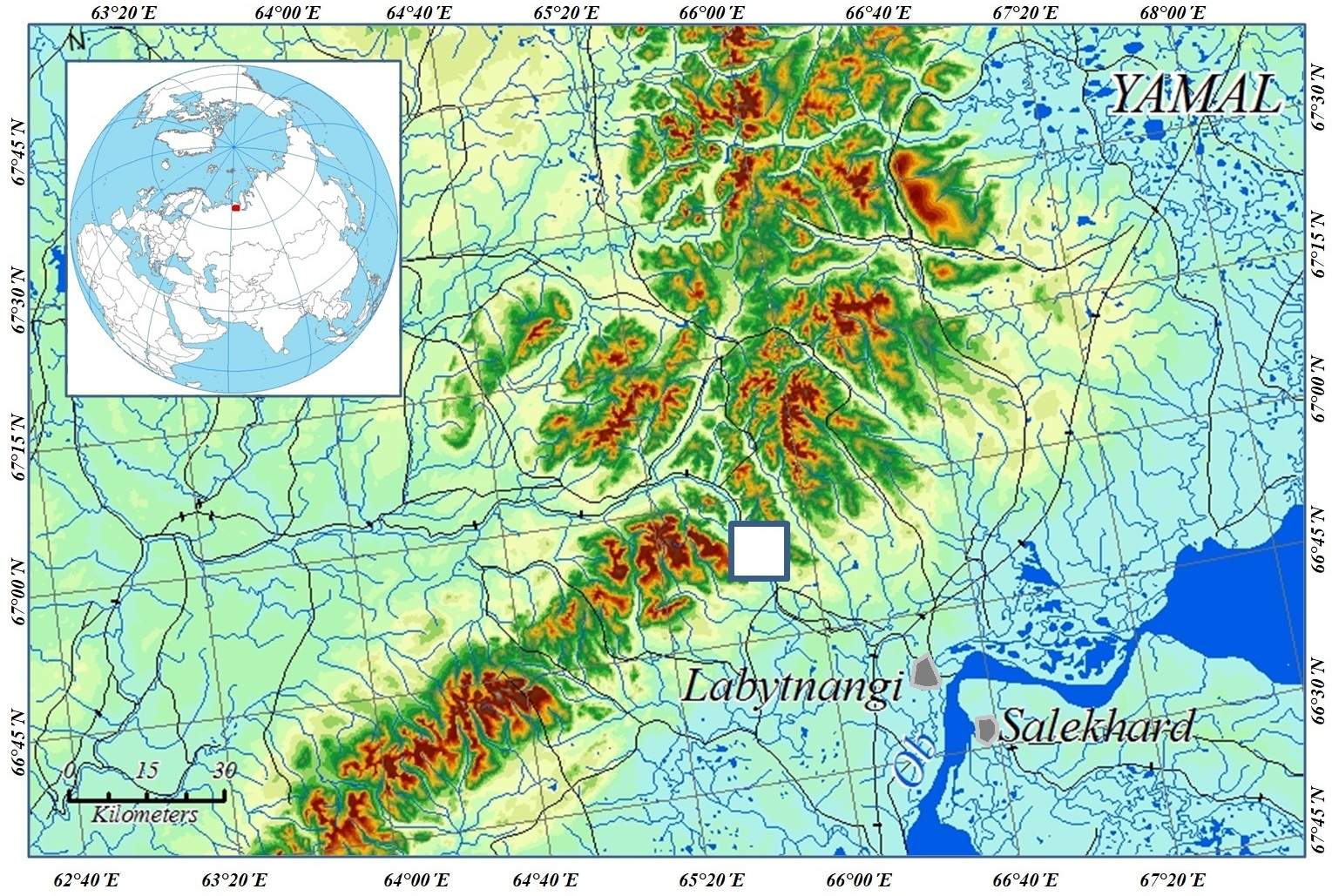

Supplement: Supplementary file 1 [file jof-06-00353-s001.zip › Figure 1.JPG]

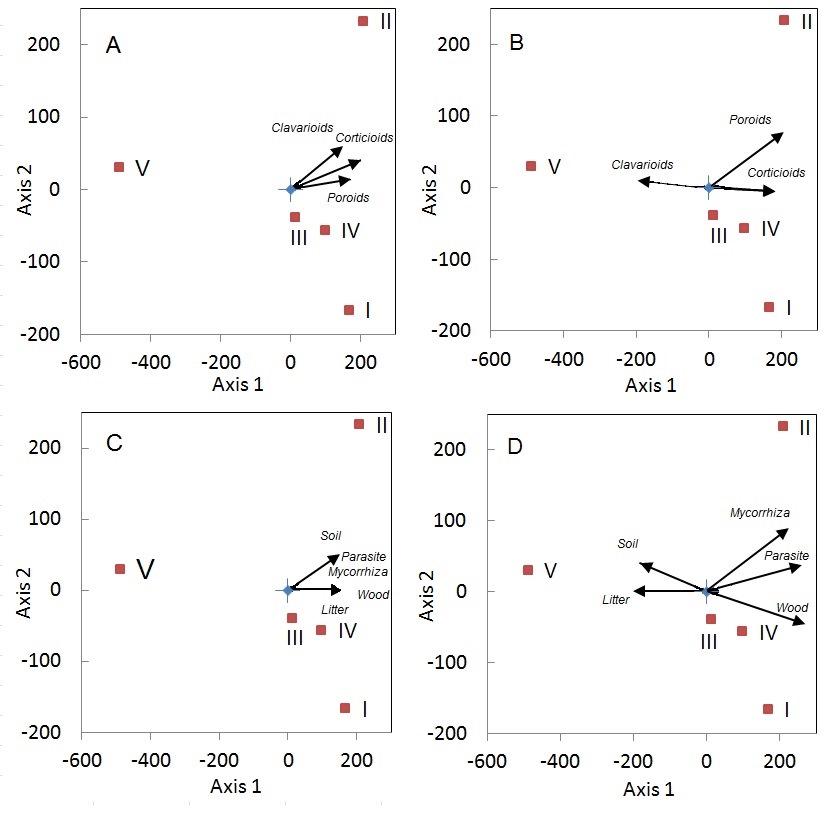

Supplement: Supplementary file 1 [file jof-06-00353-s001.zip › Figure 5.jpg]

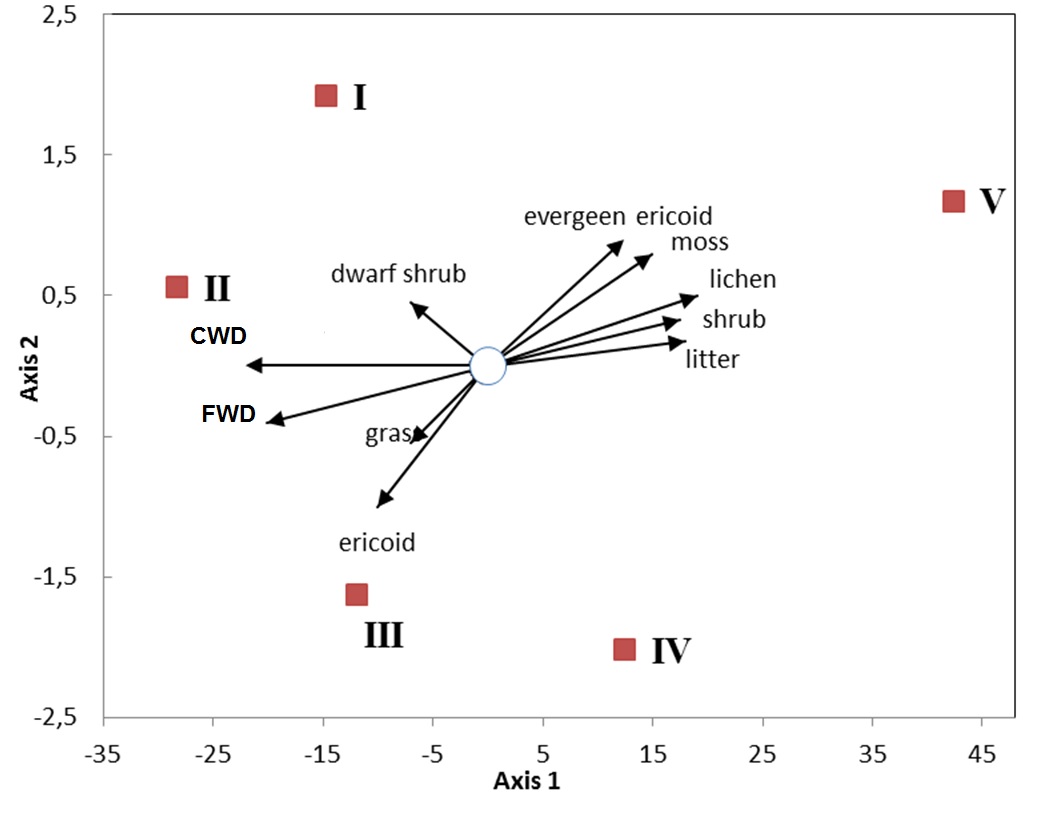

Supplement: Supplementary file 1 [file jof-06-00353-s001.zip › Figure 7.jpg]

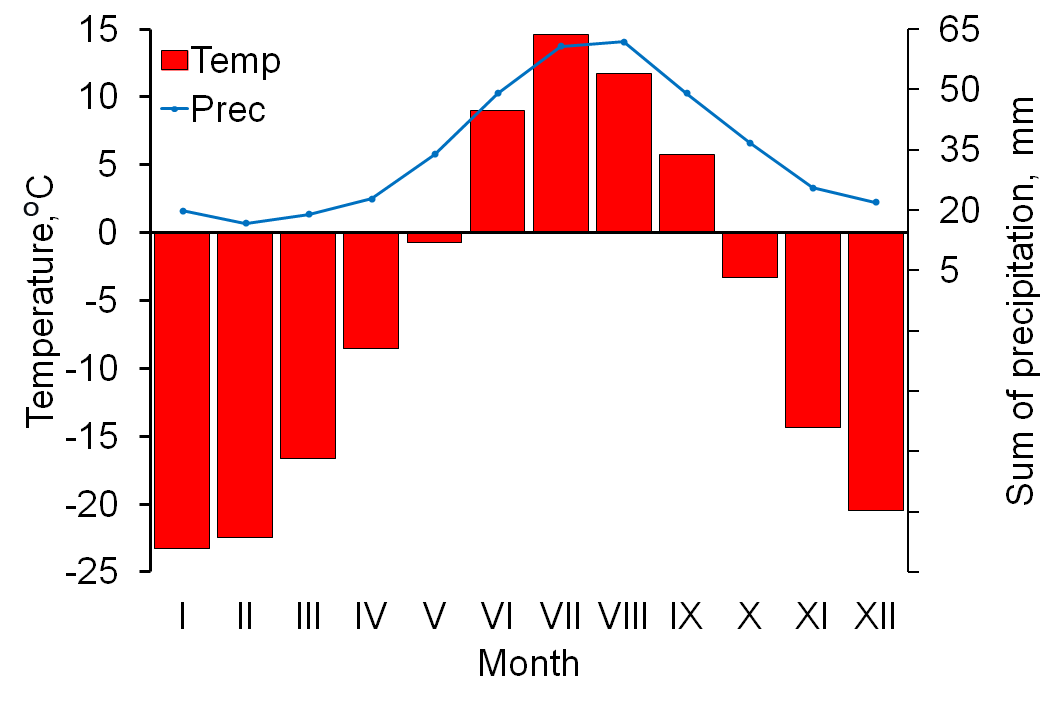

Supplement: Supplementary file 1 [file jof-06-00353-s001.zip › Figure 2_rev-1.tif]

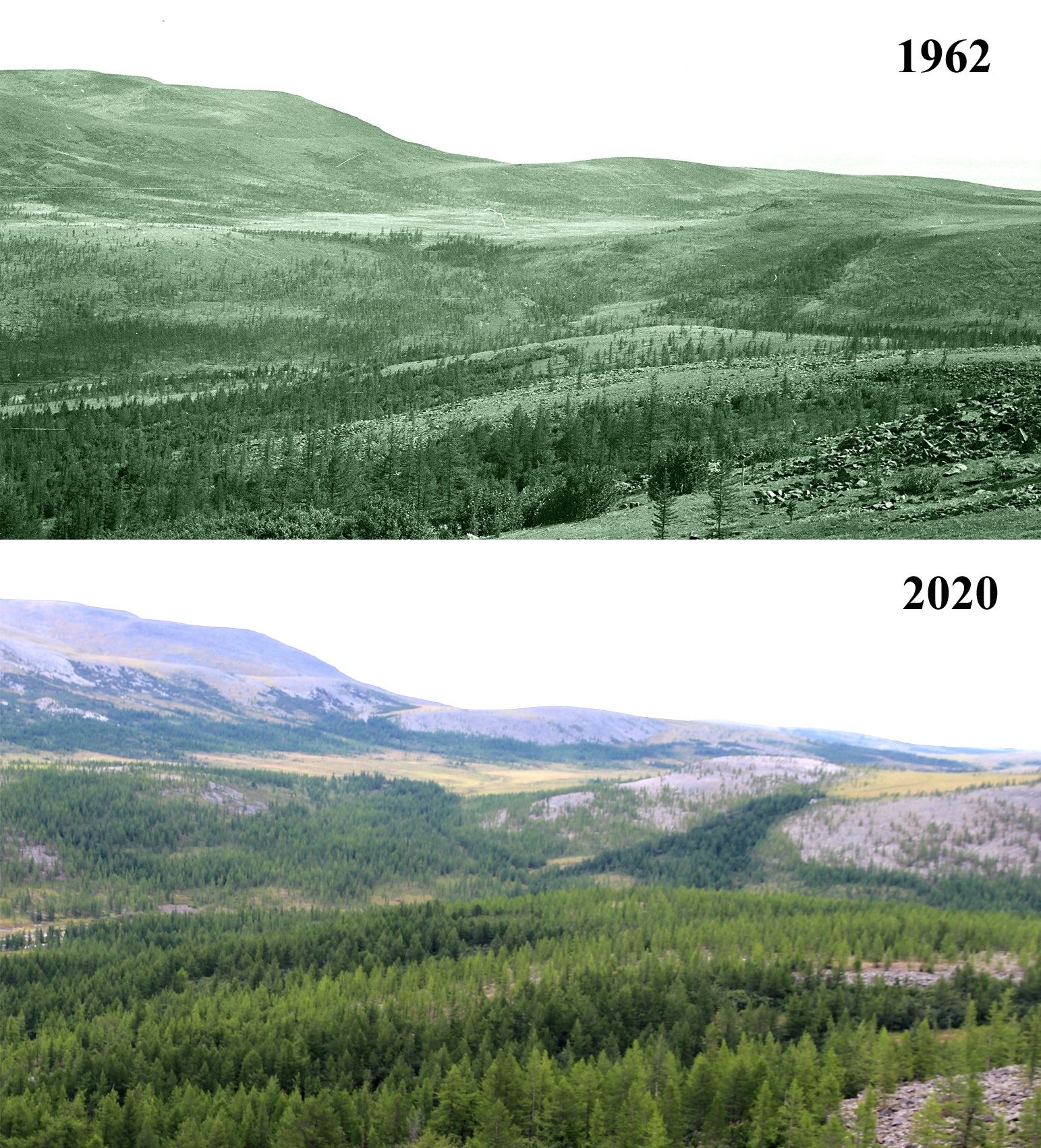

Supplement: Supplementary file 1 [file jof-06-00353-s001.zip › Figure 3.jpg]

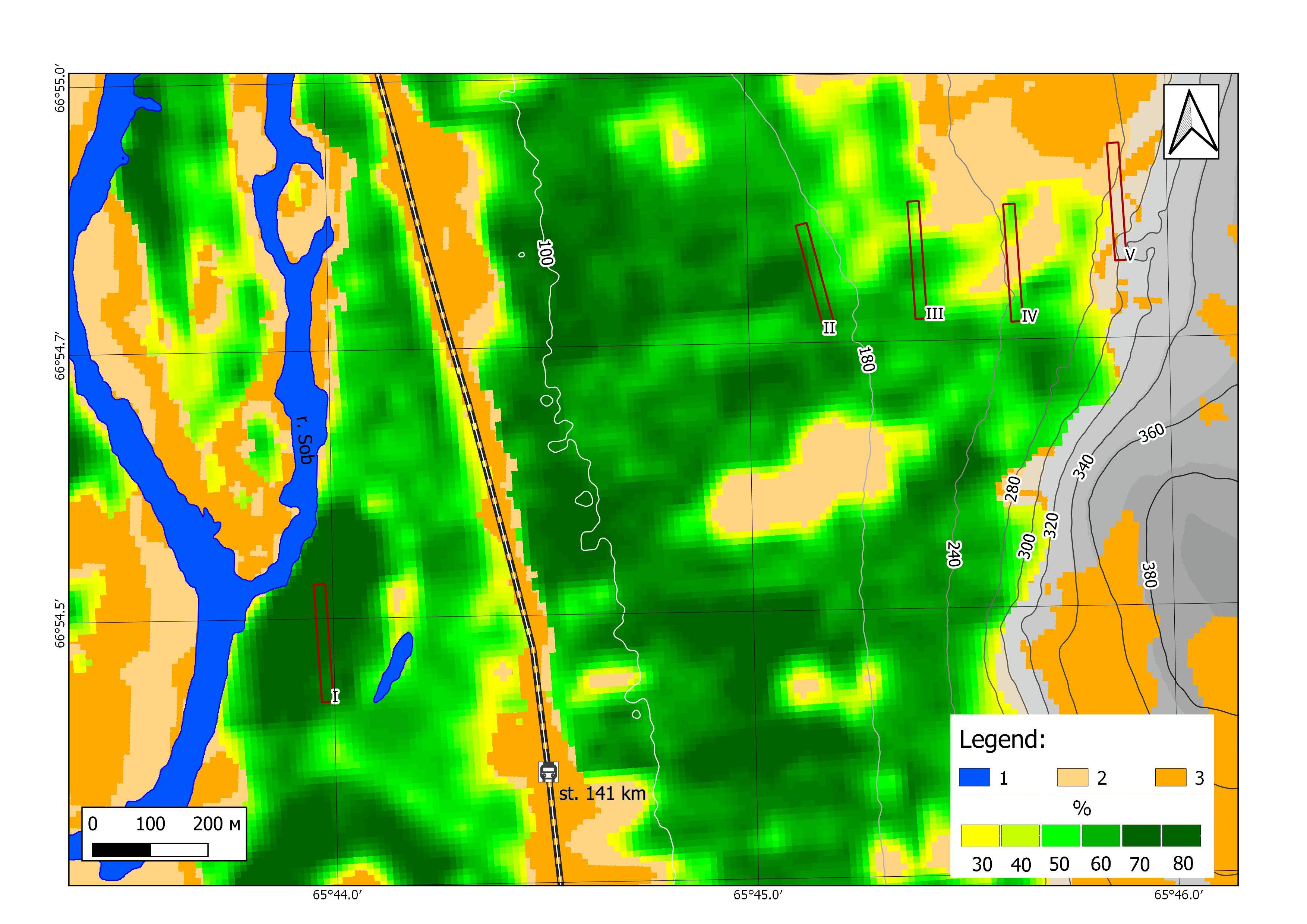

Supplement: Supplementary file 1 [file jof-06-00353-s001.zip › Figure 4.JPG]

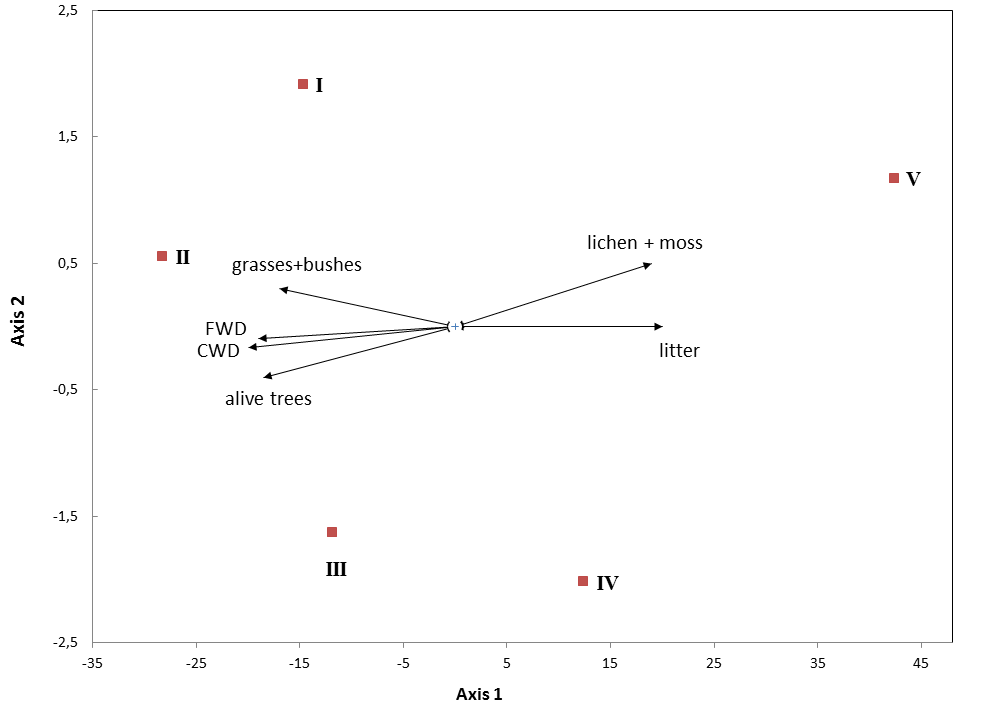

Supplement: Supplementary file 1 [file jof-06-00353-s001.zip › Figure 8.tif]

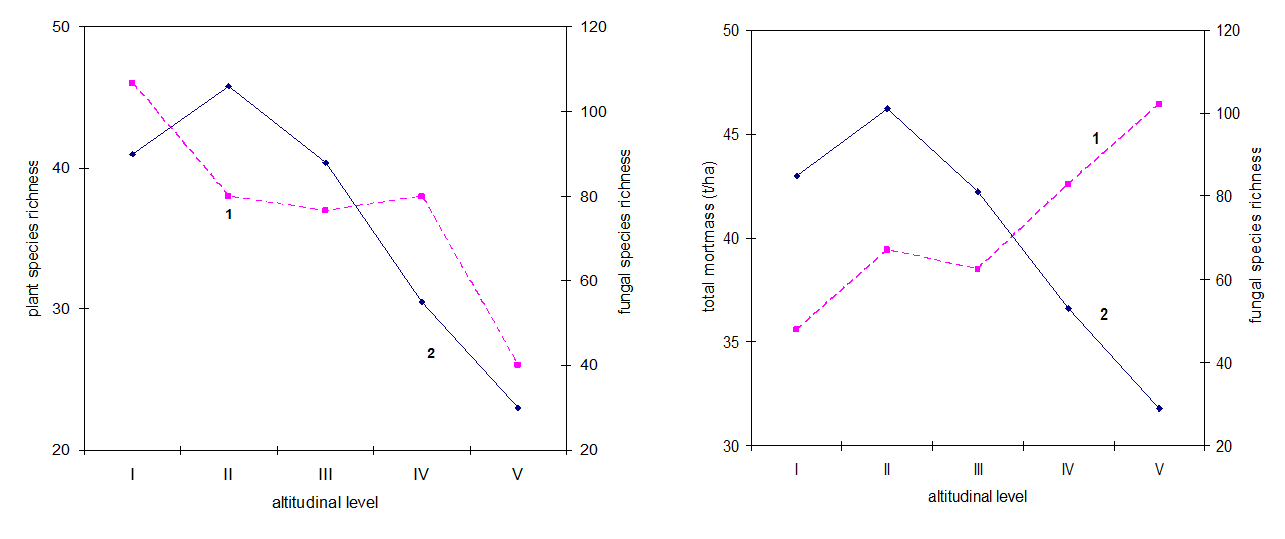

Supplement: Supplementary file 1 [file jof-06-00353-s001.zip › Figure 6.tif]
